# National-Level Consumption of Antimicrobials in the Veterinary Sector in Uganda: A Report on Analysis of Import Data for 2021

**DOI:** 10.3390/antibiotics14020150

**Published:** 2025-02-04

**Authors:** Marion Murungi, Patrick Vudriko, Helen Byomire Ndagije, Diana Nakitto Kesi, Allan Serwanga, Kalidi Rajab, Leonard Manirakiza, John Paul Waswa, Hassan Kasujja, Mark Barigye, Ddembe Kaweesi, Harriet Akello, Juliet Namugambe, Reuben Kiggundu, Niranjan Konduri

**Affiliations:** 1 USAID Medicines, Technologies, and Pharmaceutical Services (MTaPS) Program, Management Sciences for Health, Kampala P.O. Box 71419, Uganda; johnpaulwaswa@gmail.com (J.P.W.); hsnkasujja@gmail.com (H.K.); ikddembe@yahoo.com (D.K.); kajjakiggundu@gmail.com (R.K.); 2 College of Veterinary Medicine, Animal Resources and Biosecurity (COVAB), Makerere University, Kampala P.O. Box 10217, Uganda; vpato2009@gmail.com; 3 National Drug Authority, Kampala P.O. Box 23096, Uganda; hndagije@nda.or.ug (H.B.N.); dnakitto@nda.or.ug (D.N.K.); aserwanga@nda.or.ug (A.S.); mbarigye@nda.or.ug (M.B.); 4 Department of Pharmacy, Makerere University College of Health Sciences, Kampala P.O. Box 7072, Uganda; rkalidi@chs.mak.ac.ug; 5 Department of Corporate Planning, Uganda National Bureau of Standards, Kampala, P.O. Box 6329, Uganda; leonard.manirakiza@unbs.go.ug; 6 Department of Pharmaceuticals and Natural Medicines, Ministry of Health, Kampala P.O. Box 7272, Uganda; harakello@gmail.com; 7 Faculty of Infectious and Tropical Diseases, Department of Clinical Research, London School of Hygiene and Tropical Medicine, London WC1E 7HT, UK; julietsanyu@gmail.com; 8 Centers for Antimicrobial Optimization Network (CAMO-Net), Department of Global Health Security, Infectious Diseases Institute, Makerere University College of Health Sciences, Kampala P.O. Box 7072, Uganda; 9 USAID Medicines, Technologies, and Pharmaceutical Services (MTaPS) Program, Management Sciences for Health, Arlington, VA 22203, USA; nkonduri@msh.org

**Keywords:** antimicrobial resistance, antimicrobial use surveillance, antimicrobial consumption, veterinary antimicrobials, Uganda, Africa

## Abstract

Background: Antimicrobials are crucial for animal health and food security. However, their overuse in animals can lead to the emergence of resistant microorganisms. Antimicrobial resistance (AMR) poses a global public health threat that impacts both animal and human health. The objective of this study was to estimate the antimicrobial consumption (AMC) of veterinary antimicrobials at the national level using import data from January to December 2021, available from the Uganda National Drug Authority (NDA). Methods: The World Organization for Animal Health (WOAH) methodology was applied using the Anatomical Therapeutic Chemical classification codes for veterinary medicines. Results: Approximately 88,387.37 kg (88.39 tonnes) of veterinary antimicrobials were consumed in 2021. Parenteral veterinary antimicrobials accounted for 63.8% (56,375.65 kg) and oral veterinary antibacterials accounted for 36.2% (32,011.71 kg). Tetracyclines were the single most consumed veterinary antimicrobial class, accounting for 62.7% of total consumption. Oxytetracycline was the most consumed antibacterial (58.4%), followed by sulphadiazine + trimethoprim (11.1%), penicillin g/dihydrostreptomycin (7.4%), penicillin G procaine + dihydrostreptomycin (6.8%), and tetracycline (3.5%), respectively. Out of all imported veterinary antimicrobials, 76% belonged to the World Health Organization (WHO)’s Highly Important Antimicrobials (HIA) category, 16% to the Critically Important (CIA), and 9% to the Highest Priority Critically Important (HPCIA) categories. Imported colistin accounted for 0.1% of total veterinary consumption. Conclusions: This study contributes to understanding antimicrobial consumption in Uganda’s livestock sector and, for the NDA, leaves in place a system for routine surveillance at a national level. We recommend strict regulatory oversight on the importation and use of colistin and macrolides to address AMR.

## 1. Introduction

Antimicrobial resistance (AMR) is a major global public health threat. An analysis of global data showed AMR to be the third leading cause of death in 2019—4.95 million deaths were associated with, and 1.27 million deaths were directly attributable to, the phenomenon [1]. AMR disproportionately affects low- and middle-income countries (LMICs), where weak health systems, limited resources, and a high burden of infectious diseases further complicate the effective control of AMR [2,3]. The use of antimicrobials in humans, animals, food production, and the environment directly contributes to the rising rates of AMR, underscoring the importance of adopting a One Health approach to global AMR response efforts [4]. Modeling studies show that reducing antibiotic use in animal production will reduce the emergence of AMR in animals and eventually reduce the spread of resistance from animals to humans [5]. Efforts to reduce antibiotic use in food production systems and animals are ongoing [6,7,8]. However, the unavailability of data on antimicrobial consumption (AMC), especially in LMICs, presents a challenge. A systematic review of efforts to identify and combat AMR in Uganda revealed that only 12 of the 163 articles included in the analysis focused on AMR in the veterinary/animal context, and only six of the 163 articles focused on a One Health approach [9].

With over 80% of the population in Uganda engaging in agricultural activities [10] and given cited incidences of inappropriate crossover use of antimicrobials meant for humans in animals [11,12], antimicrobial-use surveillance employing a One Health approach is needed to inform the control efforts undertaken by the national AMR program. One such approach as laid out in the Uganda National Action on AMR [13] strategic objective 4 could be measuring antibiotics consumed in both human and animal health sectors. This could serve as a precursor for designing targeted interventions and informing policy and guidelines, for which data on consumption patterns at national and user levels are also needed. In this paper, we aimed to analyze the consumption of antimicrobials imported for veterinary use in Uganda using routinely collected data at the NDA.

There is still a paucity of AMC data in the veterinary sectors in the global literature. Even countries with developed surveillance systems have little information on the appropriate use of antimicrobials in animals [14]. In LMICs, data are often missing or, where available, are not truly representative of the total consumption when studies only focus on a particular species or farm level. Understanding the patterns and appropriateness of AMC in livestock at the national level is important in countries like Uganda where livestock farming and agriculture contribute to a high percentage of economic livelihood [15].

Various methodologies have been proposed to measure AMC in animals, notably, monitoring use at farm level, veterinary prescriptions and pharmaceutical distributors, and sales data. For LMICs, where sophisticated systems for data capture are not yet available, data may be gathered through Point Prevalence Studies (PPS) or longitudinal studies on consumption at the farm level; however, this would not provide a national level estimate of consumption. WHO and WOAH have recommended that countries set up surveillance systems at the national level that monitor antimicrobial consumption in both humans and animals and encourage reporting these data globally through the Global Antimicrobial Resistance and Use Surveillance System (GLASS) [16]. Using import and/or sales data is one of the recommended ways of measuring AMC at the national level [17,18]. The measurement of AMC in humans is more advanced in terms of the harmonization of methods for measurement worldwide. However, for animals, the crude measurement of antimicrobials in tonnes or kilograms indirectly represents antimicrobial exposure and is more of a measure based on selective pressure on antimicrobials.

This study presents part of the results from Uganda’s efforts to develop a national AMC surveillance system at the National Drug Authority. We used import data for the year 2021 to estimate AMC at the national level for both humans and animals. The findings of this study only focused on veterinary antimicrobials, as a report on AMC in the human sector has been published separately [19].

## 2. Results

### 2.1. Quantity of Antibacterials and Antiprotozoals per Product

Antibacterials constituted 77.2% (88,387.37 kg, or 88.39 tonnes) and antiprotozoals 22.8% (26,036.42 kg, or 26.04 tonnes) of the total antimicrobials imported for veterinary use. Among the antibacterials, oral antibacterials accounted for 36.2% (32,011.71 kg) and parenteral antibiotics for 63.8% (56,375.65 kg). Oxytetracycline, sulphadiazine + trimethoprim, and penicillin g/dihydrostreptomycin together accounted for 76.9% of antimicrobial consumption. Table 1 shows the quantities of antibacterials imported for veterinary use in 2021.

### 2.2. Proportions of Veterinary Antibacterials Imported into Uganda in 2021 by Pharmacological Class

Tetracyclines accounted for the majority (62.7%) of antibacterials imported into Uganda in 2021 (Table 2).

### 2.3. Proportions of Antibacterials Classified by WOAH Veterinary Critically Important Antimicrobials (CIA)

Almost all (99.4%) the antibacterials belonged to the WOAH Veterinary CIA class. Only a small percentage (0.6%) belonged to the WHOA Veterinary Highly Important Antimicrobial (HIA) Agents class, including colistin and ampicillin + colistin combination products.

### 2.4. Proportions of Antibacterials by WHO Critically Important Antimicrobials Classification

**Figure 1:**
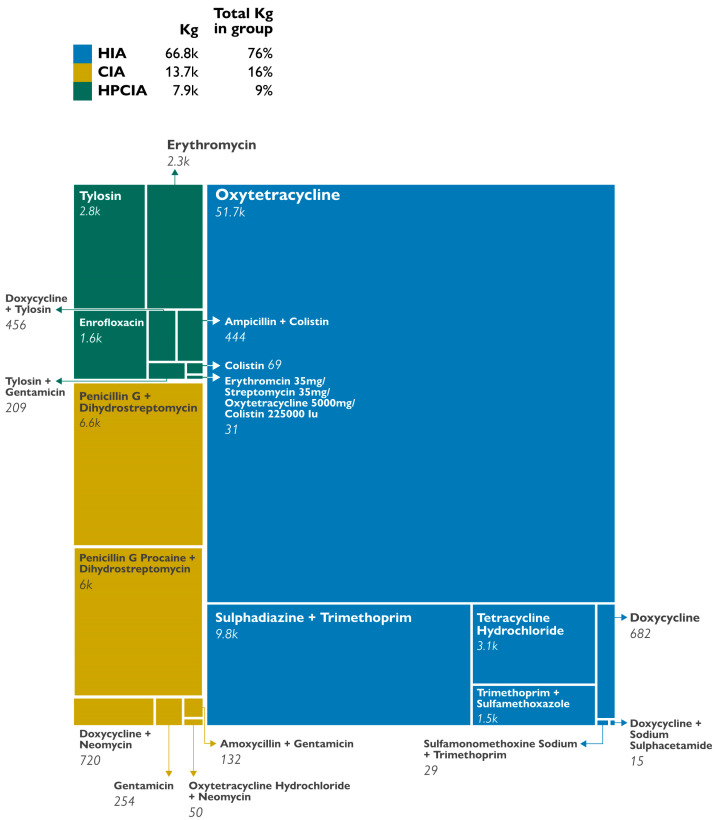
Proportions of antibacterials in each WHO classification group. HIA: Highly Important Antimicrobials; CIA: Critically Important Agents; HPCIA: Highest Priority Critically Important Agents.

Using the WHO CIA classification system, most of the antibacterials belonged to the HIA group (66,755.18 kg, 76%) and the CIA group (13,738.01 kg, 16%), with a small percentage assigned to the highest priority critically important antimicrobials (HPCIA) group (7894.18 kg, 8.9%). The HPCIA group was mostly antibacterials containing colistin and macrolides. Figure 1 shows via a Tree Map visual, the different agents in each WHO classification group.

### 2.5. Proportion by the EU AMEG Classification on Prudent Use of Antimicrobials [19]

**Figure 2:**
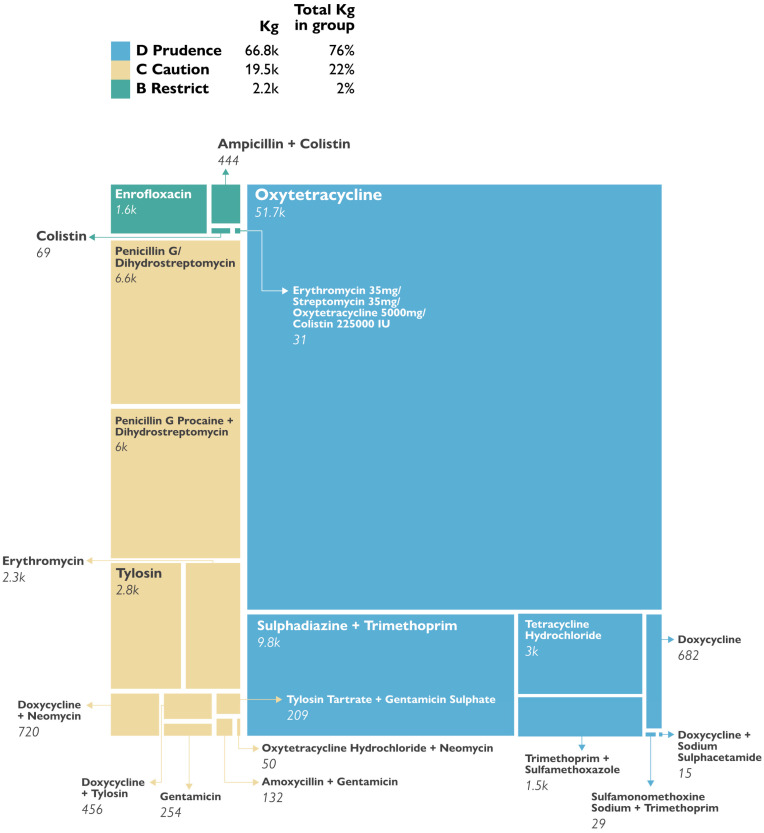
Proportions of antibacterials by EU AMEG class.

The EU AMEG classification analysis revealed that most of the antibacterials consumed belonged to the D class (low risk for AMR (66,755.18 kg, 76%), followed by C class (caution, intermediate risk) (19,472.93 kg, 22%), and finally a small proportion belonged to B class (high risk, restricted use) (2159.26, 2%). There were no medicines in the A class (avoid) imported into Uganda in 2021 (Figure 2).

## 3. Discussion

The findings of this study contribute to understanding national-level antimicrobial consumption in the veterinary sector in Uganda and is, to our knowledge, the first of such studies to come from Uganda. We demonstrate the feasibility of using national import data to measure veterinary AMC, which can be replicated in countries of similar context where point-of-use data is difficult to obtain. We present findings from a comparison of Uganda’s AMC data against the WHO, WOAH, and EU AMEG classifications and recommendations. Our study followed recommendations of the WOAH AMC surveillance in the animal health sector [20]. It also supplements another qualitative assessment and knowledge, attitudes, and practices survey conducted among veterinary drug supply chain entities and veterinary practitioners [21]. Our veterinary sector AMC study and our previous AMC studies in human health adopted a One Health approach that provides a benchmark to enable policymakers in Uganda to assess trends over time [19,22].

We found consumption to be similar to what was reported in the WOAH’s 8th Annual Report on Antimicrobial Agents Intended for Use in Animals [23] and other studies from countries in Sub-Saharan Africa [24,25,26]—that tetracyclines were the most commonly used veterinary antimicrobial, especially in low income countries, which, given the income profile, is most likely due to its affordability and broad spectrum of activity. Given the variability in consumption of antibiotics in animal health, the large number of species involved, and variations by country, it is important that national AMC data are complemented by antibiotic use patterns that will provide data on how antibiotics are used in the distribution pathway [14]. The recent development and approval of an Essential Veterinary Medicines List for Uganda [27] is a big step towards the standardization of antibiotics imported and used in the country, which in turn will strengthen surveillance and stewardship practices in the country.

The findings that oxytetracycline, sulphadiazine + trimethoprim, penicillin g/dihydrostreptomycin, penicillin G procaine + dihydrostreptomycin, and tetracycline are the most consumed antibiotics is consistent with what was observed globally (especially in LMICs) and reported in the 8th Annual Report on Antimicrobial Agents Intended for Use in Animals. This observed consumption is similar to reported patterns of use at the farm and community veterinary pharmacy levels in Uganda [28]. Additionally, the observed pattern of antibiotic consumption may not reflect use, given the reported crossover use of antibiotics meant for human health to animal health [12,29,30]. This finding has implications for antimicrobial stewardship (AMS) and One Health, with studies showing significant antibiotic residues in animal food products in Uganda [31,32]. Relatedly, the pattern of consumption at the national level will reflect use at the farm and community pharmacy levels and the rest of the distribution pathway. The poorly regulated use of antibiotics in the veterinary sector in Uganda could explain this observed pattern [12]. Recent studies have shown that most dispensers and users of veterinary drugs in Uganda do not have the necessary skills for and knowledge about veterinary medicine [21]. ‘Quick farming’ (“an entrepreneurial phenomenon that sees Ugandans raising ‘exotic’ livestock with imported methods and measures for production, including antibiotics for immediate therapy, prevention of infections and to promote production and protection of livelihoods”) as a method for growing exotic pigs and poultry with the promise of stable income contributes to the high use of antimicrobials in animals and is a major driver for AMR in Uganda [33]. As a result, these findings warrant concern because there has been an increase in the resistance of gram-negative organisms to tetracyclines (28–81%) and penicillins (42–97%), while another study reported 100% resistance of *E. coli* isolates to tetracyclines [34,35]. Rising rates of resistance to tetracyclines will be of particular concern for Uganda and other LMICs given the observed finding that tetracyclines are the most consumed veterinary antimicrobial. Very high rates of resistance of s. aureus isolates to penicillin G were reported by previous studies indicating the urgent need to translate such data into the enforcement of regulatory policies and better sensitize the animal health sector [36]. The presence of methicillin-resistant *Staphylococcus* (*S*.) *aureus* in Ugandan pig herds along with high levels of resistance to commonly used veterinary antimicrobials as found in our study further warrants continued surveillance [37]. Antimicrobial use in animals can affect resistance in humans and vice versa, further emphasizing the need for stepping up animal health sector surveillance with the overall aim to prevent AMR transmission across various One Health domains [38].

Variation was seen in antimicrobial consumption as defined by the three surveillance systems, i.e., WOAH, WHO, and EU AMEG. These systems use different nomenclatures to classify antimicrobials based on their importance for human health and the risk of resistance emergence. The findings based on WHO classification by HIA, CIA, and HPCIA now provide baseline information that can be tracked longitudinally over time. It can contribute to data-driven policy discussions to optimize the use of antimicrobials in veterinary practices via targets using a One Health approach given the risks to human health [39].

The noted finding with respect to the use of colistin in veterinary practice is of concern. The veterinary drug register for Uganda includes a number of antimicrobial combination formulations that contain colistin, which is in the category of HPCIA for use in humans and is not permitted for veterinary use in most EU countries where these formulations are manufactured. Just as it is a requirement for drugs intended for human health that a drug or antimicrobial must be licensed for use in its country of origin, there is a need for the Uganda NDA to discourage the import and registration of veterinary antibacterials (including multiple ingredient coformulations) that are not permitted for use or have been banned in their country of origin. With respect to colistin, this calls for a policy review and the activation of stricter regulatory controls because of its importance in human medicine and the high risk that resistance to this last line and potentially lifesaving antibiotic will emerge [40,41].

It is also important to note that despite ongoing efforts to set up AMC surveillance in the animal health sector [42], there remains a lack of consensus globally on the appropriate approach although the use of a common database has been applied by some LMICs to overcome this challenge [43]. Importantly, the AMC surveillance program should be developed in a gradual manner to enable systematic capacity building and, in the context of LMICs, to allow for the long-term collection of comparable data across LMICs.

One major limitation with respect to our study is that the data may have been incomplete. Another is that the biomass data on animals in Uganda that is needed to make a further detailed analysis of the use of the antibiotics were unavailable. Lastly, we could not classify consumption further by species, types of animals, etc., which may limit the interpretation and use of the findings. A further limitation of this study was that the available data did not separate out veterinary antimicrobials that come in through Uganda’s ports and are subsequently exported to neighboring countries such as South Sudan, and as such, there is a chance that our results over-estimate veterinary antimicrobial consumption. Enhanced regulatory oversight that tracks imported antimicrobials to final use and mandates importers to report on quantities sold/used within the country and those that are exported would help to overcome this limitation.

However, it is important to note that the data presented here were collected and analyzed as part of a larger objective of setting up a national surveillance system for collecting routine data at the NDA, as encouraged by WOAH. This and future iterations will contribute to improving the system, the type and quality of data it produces, and, subsequently, its useability at the country level to inform AMR policies and practices. A national level AMC/U surveillance system housed in the country’s drug regulatory body not only allows for routine surveillance and reporting but also addresses any concerns for the sustainability of AMC/U surveillance in Uganda.

Although estimating AMC based on import data is common in LMICs [25,26,44], where obtaining point-of-use data may be difficult, it risks overestimating consumption since some of what is imported may expire before it is sold or used or may be exported to neighboring countries—in the case of Uganda, to the Democratic Republic of Congo or South Sudan. Systems for AMC surveillance in Africa need to be improved with better tools, human resources capacity development, and financing. This will help improve the collection of accurate data, advance data sharing, bridge the gap between human and animal health, and strengthen data integration for a One Health approach to AMR control. This is imperative given the emerging findings in Uganda of the strong likelihood of the possible transmission of multi-drug-resistant bacteria between humans and animals [45]. It is also important to note that Uganda’s reliance on imports for veterinary medicines poses an additional risk of counterfeit, sub-standard, and or falsified drugs coming onto the market and places additional strain on the current regulatory capacity.

The findings of this study also present an opportunity for the NDA and the Ministry of Agriculture, Animal Industry and Fisheries to enforce mandatory annual reporting on imports and exports by license holders of all animal and agricultural antimicrobials such that missing data continually become more available.

In conclusion, our findings highlight the need for a national strategy on monitoring antimicrobial consumption in Uganda’s livestock sector and periodic surveillance for the emergence of resistance. We recommend strict regulatory oversight and adherence to good practices for the importation and use of colistin and macrolides to combat AMR. We propose that the Ministry of Agriculture, Animal Industry and Fisheries, in collaboration with the Ministry of Health and NDA, put in place guidance specific to the importation and use of antimicrobials, colistin, and colistin combination therapies in animals, as well as inspect veterinary importers to track endline use of antimicrobials. This will provide more information on veterinary practices, especially those crucial to AMR and the emergence of resistance.

Future research should consider linking national veterinary AMC to animal demographics in Uganda using a Population Correction Unit as recommended by WOAH to ease international comparisons of veterinary AMU.

## 4. Materials and Methods

### 4.1. Study Setting

Uganda is a landlocked country in East Africa with a population of 48.8 million people [46]. Uganda’s Gross Domestic Product (GDP) per capita is USD 607 annually, with the agricultural sector contributing 24.6% to the GDP and accounting for 71% of employment [47]. Most (95%) of the medicines used in the country, including those used in animal production and agriculture, are imported. All imports of medicines and pharmaceuticals are regulated by the National Drug Authority (NDA). Veterinary medicines are distributed mainly through the private sector, with importers and wholesale pharmacies selling to retail outlets (pharmacies, drug shops) that in turn sell to farmers.

### 4.2. Study Design and Data Sources

We conducted a retrospective review of records of all veterinary antimicrobials imported into the country in 2021. Access to the data was sought from and granted by the NDA, and records were accessed through the NDA management information system.

### 4.3. Data Collection

Using a web-based tool developed by the NDA to extract the variables required for measuring antimicrobial consumption (AMC) from the NDA management information system into a Microsoft Excel^®^ version number 2312 dataset, we obtained a list of all antimicrobials (human and veterinary) imported into the country. For accuracy, we cross-checked this with paper import documents from the four different ports of entry/customs (Nakawa, Entebbe, Malaba, and Busia) where medicines enter the country and are verified by the NDA before being introduced into the supply chain distribution. The variables in the Microsoft Excel^®^ version 2312 dataset included NDA registration number, brand name, generic name, active pharmaceutical ingredients, route of administration, dosage form, strength and unit of measure, pack size and unit of measure, batch quantity, unit cost, total cost, NDA verification fees paid, currency used, import transportation details, country of manufacture, license holder, date of entry, year of import, country of origin, country of manufacture, name of importer, and type of import (human or veterinary). For purposes of calculating AMC, we also collected data on strength per pack, base quantity, total quantity imported in grams, total quantity in kilograms, Defined Daily Dose (DDD), Anatomical Therapeutic Chemical Veterinary (ATCvet) classification code [48], and human Anatomical Therapeutic Chemical (ATC) classification code. For data verification, all entries were cross-checked with the NDA veterinary drugs registers (January and July 2021 versions). For missing data, we tracked individual corresponding paper records, and or cross-checked with the drug register to fill the gap. We were unable to include products locally manufactured within the country because information on those was not readily available in NDA records. The active ingredients are imported, and data regarding these imports are recorded at the port of entry, which is reflected in our import data.

### 4.4. Antimicrobials Included

For this study, we included veterinary antimicrobials belonging to the following ATCvet classes: QJ01—antibacterials for systemic use; QJ51—antimicrobials for intramammary use; QG01—gynecological anti-infectives and antiseptics; QA07A—intestinal anti-infectives; and QG51— anti-infectives and antiseptics for intrauterine use.

### 4.5. Data Analysis

For this analysis, we adopted the World Organization for Animal Health (WOAH) methodology for calculating the quantities of antimicrobials used [49]. For calculation of kilograms (kg) of antimicrobial agent (active chemical ingredient), the stated amount on each package was used and converted from mg, international units, weight-in-volume, or weight-in-weight to grams. The total grams per package was multiplied by the total number of packages imported that year to get the annual consumption, which was then divided by 1000 to obtain quantity in kg.

$$\mathrm{Unit}\;\mathrm{annual}\;\mathrm{AMC}\;(\mathrm{kg})=\frac{\mathrm{Amount}\;\mathrm{of}\;\mathrm{each}\;\mathrm{package}\;\mathrm{used}\;\left(g\right)\;\times \;\mathrm{total}\;\mathrm{number}\;\mathrm{of}\;\mathrm{packages}\;\mathrm{imported}}{1000}$$

For analysis, drugs were categorized by pharmacological, WHO Critically Important Antimicrobials (CIA), WOAH Veterinary Critically Important Antimicrobial Agents (VCIA), and the European Union’s Antimicrobial Advice Ad-Hoc Expert Group (AMEG) classifications [50].

The mathematical conversions were performed in a Microsoft Excel^®^ version 2312 sheet and transferred to IBM SPSS^®^ version 28 for analysis. Quantities were summarized as proportions.

## Figures and Tables

**Table 1:** Quantity (kg) of antibacterials intended for veterinary use imported into Uganda in 2021.

			Quantity in Kg	
	* VET_ATC5 CODE	Medicine Name	Kg	%
1	QJ01AA06	Oxytetracycline	51,657.21	58.4%
2	QJ01EW10	Sulphadiazine + Trimethoprim	9776.54	11.1%
3	QJ51RC22	Penicillin G + Dihydrostreptomycin	6564.02	7.4%
4	QJ51RC23	Penicillin G Procaine + Dihydrostreptomycin	6017.80	6.8%
5	QJ01AA07	Tetracycline Hydrochloride	3050.02	3.5%
6	QJ01FA90	Tylosin	2818.08	3.2%
7	QJ01FA01	Erythromycin	2251.35	2.5%
8	QJ01MA90	Enrofloxacin	1615.51	1.8%
9	QJ01EW11	Trimethoprim + Sulfamethoxazole	1545.89	1.7%
10	QA07AA51	Doxycycline + Neomycin	720	0.8%
11	QJ01AA02	Doxycycline	682	0.8%
12	QJ01RA90	Doxycycline + Tylosin	456.4	0.5%
13	QG51AG07	Ampicillin + Colistin	444	0.5%
14	QJ01GB03	Gentamicin	254.12	0.3%
15	Not assigned	Tylosin Tartrate + Gentamicin Sulphate	209.09	0.2%
16	QJ01RA01	Amoxycillin + Gentamicin	131.67	0.1%
17	QA07AA10	Colistin	69.03	0.1%
18	QJ01AA56	Oxytetracycline + Neomycin	50.4	0.1%
19	QJ01RA95	Erythromcin 35 mg/Streptomycin 35 mg/Oxytetracycline 5000 mg/Colistin 225,000 IU	30.72	0.0%
20	QJ01EW17	Sulfamonomethoxine Sodium + Trimethoprim	28.5	0.0%
21	QJ01RA90	Doxycycline + Sodium Sulphacetamide	15.02	0.0%
		Total	88,387.37	100.0%

* VET_ATC5: Veterinary Anatomical Therapeutic Chemical Level 5 Category.

**Table 2:** Proportions by pharmacological class of antibacterials imported in Uganda in 2021.

				TOTAL QTY IN KG	
	ATC3 Code	ATC3Name	ATC4Name	Kg	%
1	QJ01AA	Tetracyclines	Tetracyclines	51,657.21	59.0%
2	QJ51R	Combinations of antibacterials for intramammary use	Aminoglycoside + Penicillin	9776.54	11.2%
3	QJ01E	Sulfonamides and trimethoprim	Sulfonamides and trimethoprim	6564.02	7.5%
4	QJ01F	Macrolides	Macrolides	6017.80	6.9%
5	QJ01M	Fluoroquinolones	Fluoroquinolones	3050.00	3.5%
6	QA07A	Intestinal antiinfectives	Aminoglycoside + Tetracycline	2818.08	3.2%
7	QJ01R	Combinations of antibacterials	Tetracycline + Macrolide	2251.35	2.6%
8	QG51A	Antiinfectives for intrauterine use	Polymixin + Penicillin	1615.51	1.8%
9	QJ01G	Aminoglycosides	Aminoglycosides	1545.89	1.8%
10	QJ01R	Combinations of antibacterials	Aminoglycoside + macrolide	720	0.8%
11	QA07A	Intestinal antiinfectives	Polymixins	682	0.8%
12	QJ01R	Combinations of antibacterials	Polymixin + three others	456.4	0.5%
13	QJ01R	Combinations of antibacterials	Tetracyclines + Sulfonamides	444	0.5%
			TOTAL	87,598.80	1.00

## Data Availability

Data will be made available upon request.
